# HormoNet: a deep learning approach for hormone-drug interaction prediction

**DOI:** 10.1186/s12859-024-05708-7

**Published:** 2024-02-28

**Authors:** Neda Emami, Reza Ferdousi

**Affiliations:** Department of Health Information Technology, School of Management and Medical Informatics, Tabriz University of Medical Sciences, Tabriz, Iran

**Keywords:** Hormone–drug interaction, Deep learning, Interaction prediction, Hormone interaction

## Abstract

Several experimental evidences have shown that the human endogenous hormones can interact with drugs in many ways and affect drug efficacy. The hormone drug interactions (HDI) are essential for drug treatment and precision medicine; therefore, it is essential to understand the hormone-drug associations. Here, we present HormoNet to predict the HDI pairs and their risk level by integrating features derived from hormone and drug target proteins. To the best of our knowledge, this is one of the first attempts to employ deep learning approach for prediction of HDI prediction. Amino acid composition and pseudo amino acid composition were applied to represent target information using 30 physicochemical and conformational properties of the proteins. To handle the imbalance problem in the data, we applied synthetic minority over-sampling technique technique. Additionally, we constructed novel datasets for HDI prediction and the risk level of their interaction. HormoNet achieved high performance on our constructed hormone-drug benchmark datasets. The results provide insights into the understanding of the relationship between hormone and a drug, and indicate the potential benefit of reducing risk levels of interactions in designing more effective therapies for patients in drug treatments. Our benchmark datasets and the source codes for HormoNet are available in: https://github.com/EmamiNeda/HormoNet.

**Supplementary Information:**

The online version contains supplementary material available at 10.1186/s12859-024-05708-7.

## Introduction

Recent reports have shown that the human endogenous hormones can interact via drugs in many ways and significantly affect drug efficacy. The interactions between hormones and drugs are crucial for drug treatment and precision medicine, therefore it is essential to understand the hormone-drug associations. For instance, in an experiment to examine the effect of stress hormones on the efficacy of a microtubule disrupting agent, paclitaxel, in co-culture through Cdk-*1* in breast cancer cell lines (MDA-MB-*231*), it resulted that stress hormones have negative affections [1]. In another study, they showed that stress hormones (cortisol, norepinephrine, and epinephrine) can render drug resistance to paclitaxel, which may have profound implications for the treatment of drug resistance in patients with Triple-negative breast cancer [2].

However, only a limited number of hormone-drug pairs among the large number of hormones and drugs have been studied so far. Most previous studies have focused only on certain types of hormones (stress hormones) and drugs (cancer-treating drugs). Therefore, in order to better understand the relationships between hormones and drugs, it is necessary to investigate other types of hormones and drugs pairwise.

Most of the previous studies in this scope have been conducted through in-vivo and in-vitro process-based methods, especially using cell lines, since mainly the drugs studied were cancer treatment drugs. In-vivo and in-vitro methods are accurate and reliable, but they are not appropriate for analyzing whole pairwise combinations of hormones and drugs since these processes are challenging, time-consuming, and often require high costs. Computational methods can accelerate the process of testing whole pairwise combinations of hormones and drugs and save cost.

To the best of our knowledge, there are two in silico-based approaches have been developed so far for hormone and drug study. In [3], Sun et al. proposed a model to uncover how epinephrine affects apoptosis-regulating mechanisms of eight prostate cancer drugs, using ordinary differential equations. They found that epinephrine signaling interfered with apoptosis induced in prostate cancer cells by combinations of signal transduction inhibitors. Consequently, this process decreases the chemotherapeutic efficacy of prostate cancer drugs. The quantitative models’ parametric characteristics such as ODE models facilitate accurate network analysis however require optimizing of several parameters. In another study [4], Kwon et al. proposed a predictor based on hormone effect paths and drug effect paths using a scoring function to define hormone impacts on drug efficacy. Although, their predictor had yielded favorable results, however there are several opportunities and requirements for enhancing this field.

The use of the deep learning approaches as powerful tools have had a high performance in biological problems [5–9], and they have not yet been applied as a computational tool for prediction of hormone–drug interactions. To this purpose, we leveraged a novel conventional neural network (CNN)-based approach to predict HDI pairs and possible their risk level based on 30 physicochemical and conformational properties of hormone receptors and drug targets information. To handle the imbalance problem in our dataset, we used a data augmentation procedure [10]. Building on this contribution, here we presented a novel CNN-based approach for HDI prediction and the possible their risk level. The system is called ‘HormoNet’ for ease of reference. The use of HormoNet goes beyond previous work as it uses a deep learning method for prediction and achieved high performance on our constructed benchmark dataset.

## Results

This section summarizes the outcomes of several evaluation experiments on our model.

### The results of data collection

In order to construct our reliable datasets, we collected following data from six different databases: Human endogenous hormones and their receptors from EndoNet, Drug-drug interactions from DDInter, Drug-target associations from DrugBank, Protein sequences from UniProtKB/Swiss-Prot, Protein–protein interactions from BioGRID and TRI-tool (see Materials and methods).

First, we obtained drug-drug interactions and clarified a relation for every drug-drug interaction (see “Materials and methods”). Second, Hormones and drugs should have at least one protein receptor or protein target, respectively.

As a result, *283* human hormones and *451* receptors have been extracted. A total number of *8961* drug-drug interactions have been found. Additionally, *2209* drug-protein target associations have been found. For protein–protein interaction; *9230* interactions containing *4773* positive and *4457* negative interactions have been obtained.

Finally, for constructing the first stage’s dataset, the total number of instances were *9230*, which contain *4773* positive and *4457* negative instances, include *28* hormones, *443* drugs and 28 hormone receptors and *321* protein targets were obtained. For building the second stage’s dataset, the *4773* interactions containing risk levels have been considered include *21* hormone, *20* hormone receptors, *312* drugs and *295* protein targets were obtained.

### The results of the balancing dataset

This study intends to provide an accurate approach to identify the risk levels of HDIs. The data imbalanced problem resulted in inefficient training of the predictors on the minority class, i.e., moderate. This resulted in a higher proportion of test samples incorrectly predicted from the target variable corresponding to moderate level. To deal with this problem, SMOTE was implemented to obtain a balanced dataset for effective training of our model. Table 1, presents the number of samples that each class had before and after applying SMOTE.

**Table 1:** Number of instances for each class before and after applying SMOTE technique

	Before SMOTE				After SMOTE			
	Samples	Class A	Class B	Class C	Samples	Class A	Class B	Class C
All	*4773*	*561*	*3701*	*511*	*11,103*	*3701*	*3701*	*3701*
Train	*3579*	*429*	*2778*	*372*	*8327*	*2785*	*2765*	*2777*
Test	*1194*	*132*	*923*	*139*	*2776*	*916*	*936*	*924*

The number of samples in each classes in the data set is not the same, therefore this SMOTE balanced the number of samples in all classes are highlighted in italic

For risk level of interaction of drug A and drug B: class A is major, class B is moderate, and class C is minor

Compared with the condition of original unexpanded dataset and the data expanded by the SMOTE algorithm, it is clear that the performance of our proposed model is boosted after applying SMOTE, which shows an increase in classification; accuracy increased by *0.0494*; Precision increased by *0.0819*; Recall increased by *0.2407*; and F1-score increased by 0.2705, for training dataset. And, accuracy increased by 0.0308; Precision increased by *0.0011*; Recall increased by *0.2149*; and F1-score increased by *0.2348*, for testing dataset.

Table 2 shows the performance of our proposed model before and after applying SMOTE technique, respectively.

**Table 2:** Our model’s performance before and after applying SMOTE technique

Model		Accuracy	F1-Score	Precision	Recall
Train	*Before*	*0.7773*	*0.3570*	*0.7009*	*0.3682*
	*After*	*0.8267*	*0.6275*	*0.7828*	*0.6089*
Test	*Before*	*0.7781*	*0.3567*	*0.7130*	*0.3690*
	*After*	*0.8089*	*0.5915*	*0.7141*	*0.5839*

Since deep learning methods require high volume of data, therefore the performance of deep learning methods increases with the increase in the number of samples. In our study, since SMOTE increased the number of samples in the dataset by balancing the number of samples in each classes, thus the performance of the model after applying SMOTE has been increased and are highlighted in italic

### The results of deep neural networks performances

**Fig. 1:**
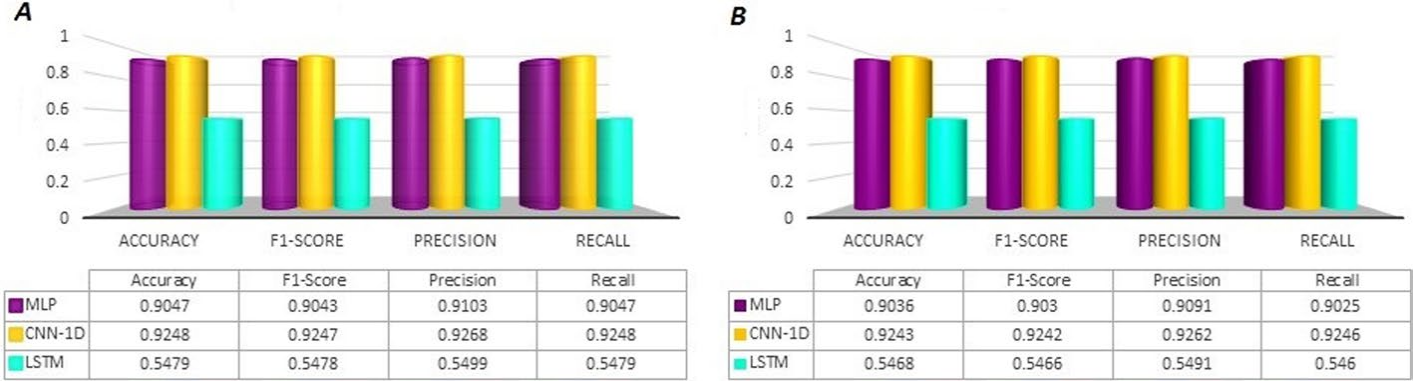
Comparison of the prediction performances of three deep neural network on our benchmark dataset, where **A** and **B** show results for train test, respectively. MLP: multilayer perceptron, CNN-1D: convolutional neural network, and LSTM: long short term memory

In order to select the appropriate deep neural network for prediction of HDI, we compared three deep neural networks: Multy layer perceptron (MLP), CNN, and long short term memory (LSTM). To set the neural networks we used the following: rmsprop algorithm was considered as an optimizer with its default values; the number of batch sizes: 16; and epochs: 30. Figure 1 depicts the outcomes in terms of accuracy, recall, precision, and F1-score gained by MLP, CNN, and LSTM.

It is evident that CNN provides the highest performances for our benchmark dataset. Therefore, we selected CNN as our classifier. These outcomes have demonstrated the competitive performance of CNN in predicting HDI.

### The results of the CNN optimization

**Fig. 2:**
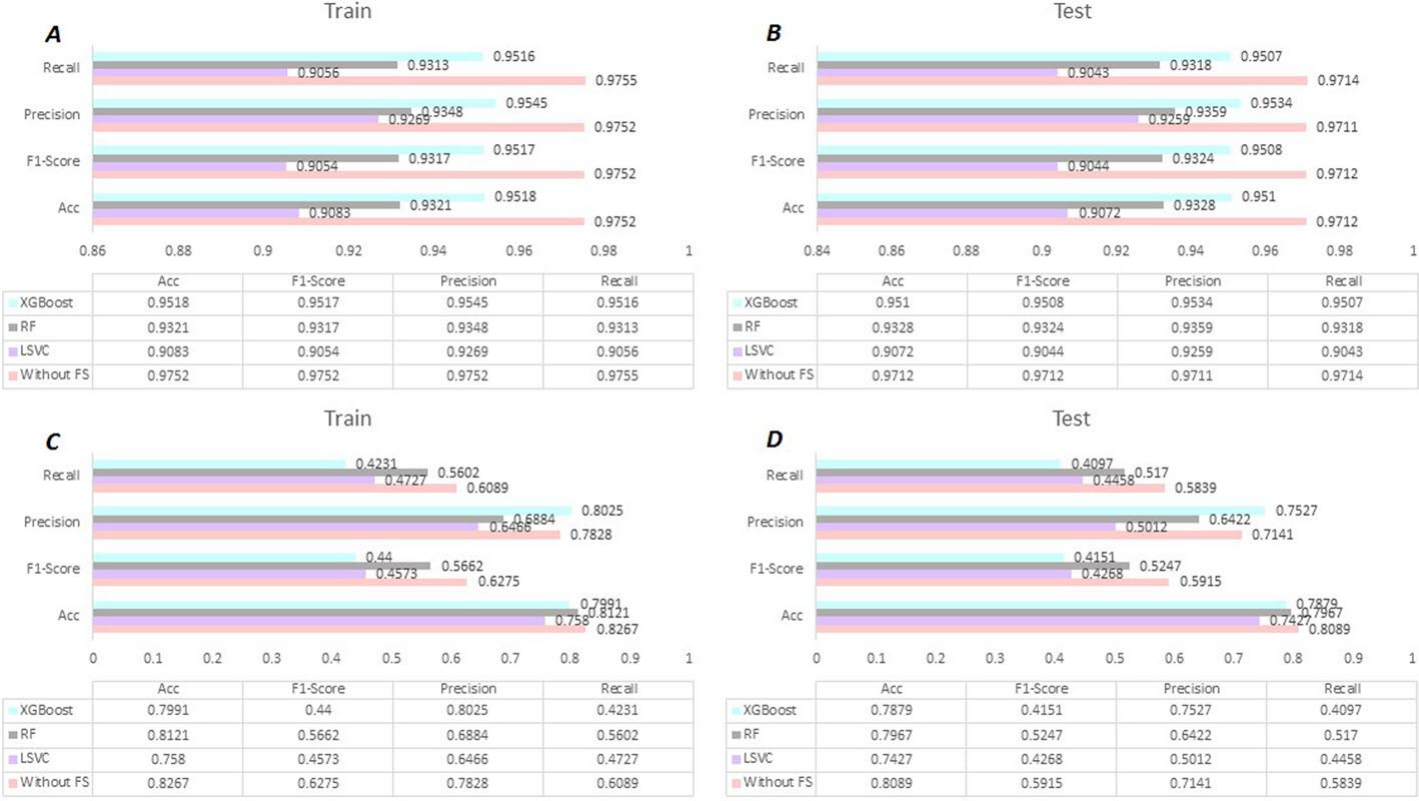
The results of HormoNet on our benchmark datasets for hormone-drug interaction and risk level before and after applying feature selection strategies. RF: Random Forest, LSVC: Linear Support Vector Classification, and XGBoost: eXtreme Gradient Boosting

According to Fig. 1, it is evident that CNN provides the highest performances for our benchmark dataset. Therefore, we selected CNN as our classifier. To improve the performance and adjust the optimal state, the diverse hyperparameter for proposed model were implemented. The final values are as follow: Epochs = 50, Learning rate = 0.00025, and batch size = 16. In this study we have applied three different strategies including Random Forest (RF), Linear Support Vector Classification (LSVC), and eXtreme Gradient Boosting (XGBoost) techniques to select most important features. However, the outcomes of our predictor have not improved after applying feature selection (see Fig. 2), therefore we have presented our final model without feature selection.

For RF, the parameters were set based on our several feature selections experiments. The estimator’s value was set *300* and max depth value was set *9* based on our feature selection experiments. The dimensions of our initial datasets were 9230*900 and 7963*900 for HDI prediction and risk level prediction, respectively. This method reduced the number of features. In other words, this method reduced the dimensions of the dataset from 9230*900 to 9230*162 for HDI and 7963*900 to 7963*324 for HDI risk level. For LSVC, the parameters were set based on our several feature selections experiments. The penalization was based on *l2* norm and estimator value was set *300*. This method reduced the dimensions of the dataset from 9230*900 to 9230*293 for *HDI* and 7963*900 to 7963*337. For XGBoost, the parameters with its default values. This method reduced the dimensions of the dataset from 9230*900 to 9230*177 for HDI and 7963*900 to 7963*259. However, the outcomes of our predictor have not improved after applying feature selection techniques (see Fig. 2).

**Fig. 3:**
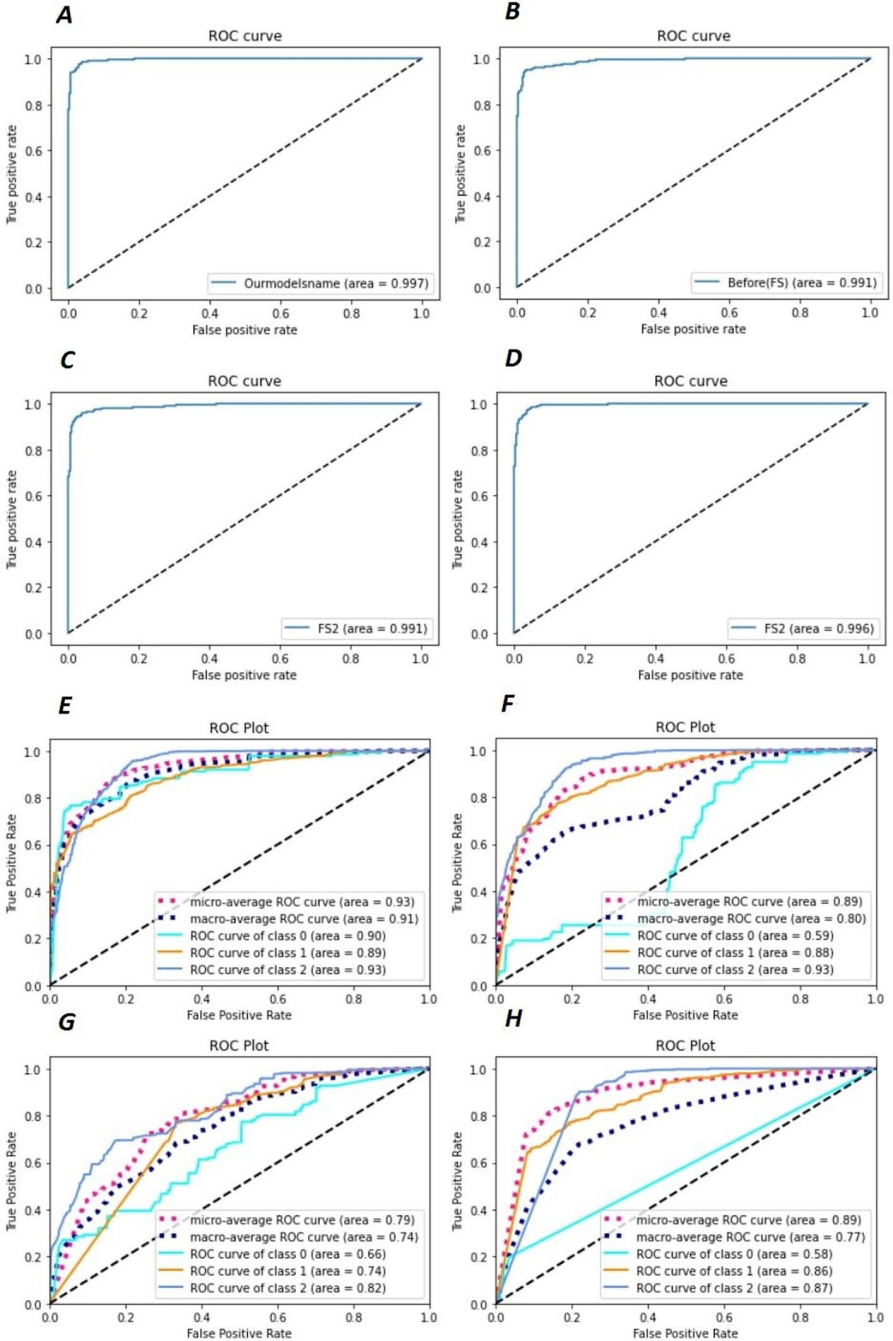
Receiver operating characteristic (ROC) curves of HormoNet before and after feature selection techniques on our benchmark datasets. Where A depicts the prediction performance of HormoNet for HDI before using feature selection, B illustrates the prediction performance of HormoNet for HDI after using RF, C shows the prediction performance of HormoNet for HDI after using lsvc, D is the prediction performance of HormoNet for HDI after using XGBoost.E is the prediction performance of HormoNet for risk level before applying feature selection methods, F is the prediction performance of HormoNet for risk level after RF, G is F is the prediction performance of HormoNet for risk level after lsvc, and H is F is the prediction performance of HormoNet for risk level after XGBoost

**Fig. 4:**
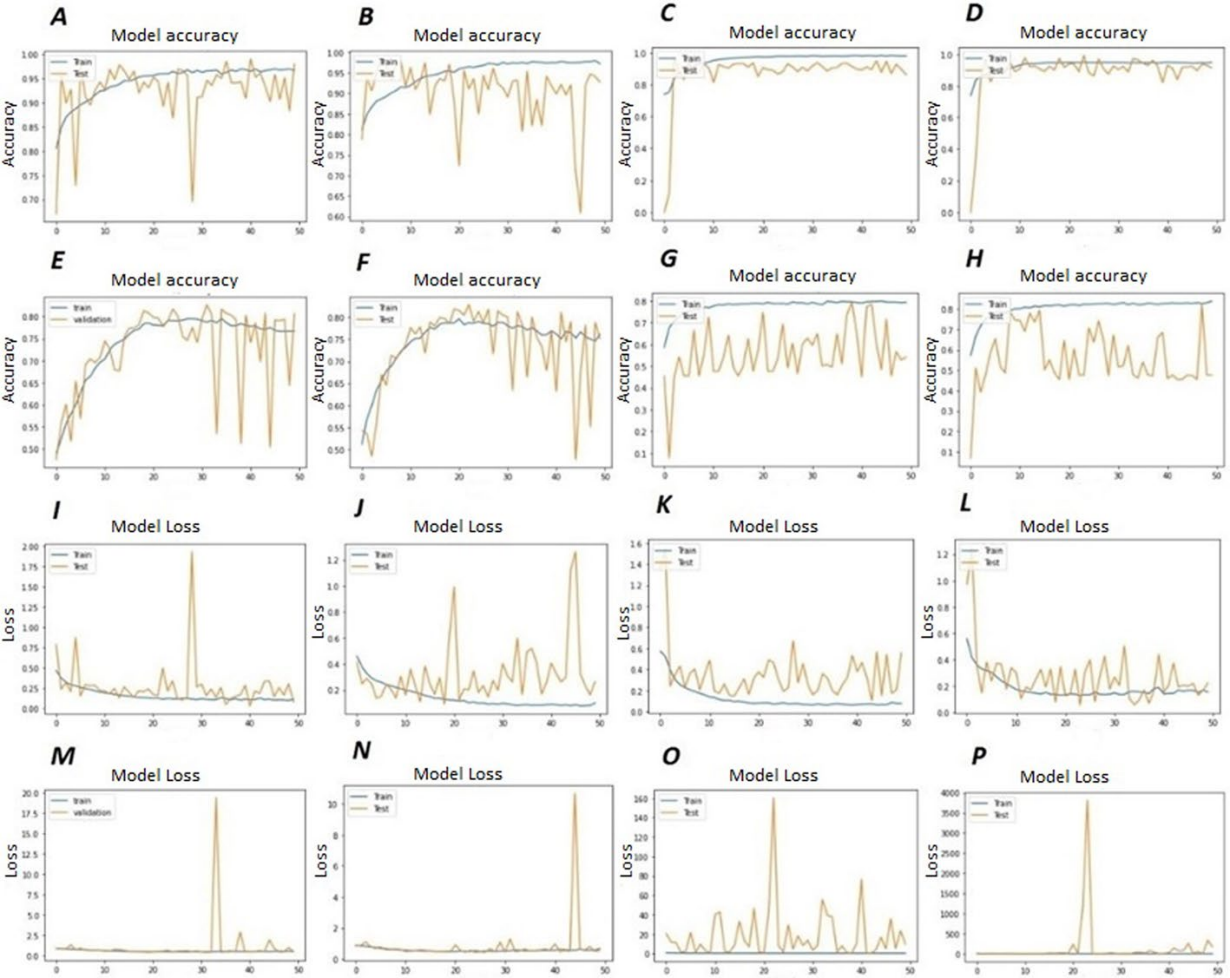
Model accuracy and loss of HormoNet before and after feature selection techniques on our benchmark datasets. Where, **A** is the model accuracy of HormoNet for HDI before using feature selection, **B**, **C**, and **D** are model accuracy of HormoNet for HDI after using RF, lsvc, and XGBoost. **E** is model accuracy of HormoNet on our benchmark dataset for hormone-drug interaction risk level. **F**, **G**, and **H** are model accuracy of HormoNet for risk level after RF, lsvc, and XGBoost. **I** is the model loss of HormoNet for HDI before using feature selection, **J**, **K**, and **L** are model loss of HormoNet for HDI after using RF, lsvc, and XGBoost. **M** is model loss of HormoNet on our benchmark dataset for hormone-drug interaction risk level. **N**, **O**, and **p** are model loss of HormoNet for risk level after RF, lsvc, and XGBoost.

Figure 3 illustrates the ROC plots for HormoNet and Fig. 4 shows model accuracy and loss of HormoNet for batch size = 16 and epoch = 50. According to Fig. 4, the ROC values for HormoNet had not improved after applying feature selection.

Figure 3 presents ROC values of HormoNet on our benchmark dataset for prediction of HDI and their risk level before and after applying FS strategies. As it is clear, the performance of our model did not improve after applying three different FS methods.

Figure 4 illustrates the model accuracy and loss of HormoNet on our benchmark dataset for prediction of HDI and their risk level before and after applying FS strategies. It is clear that, for prediction of HDI and their risk level by HormoNet, model accuracy has been decreased and model loss has been increased. These outcomes have demonstrated the competitive performance of hormonet in predicting HDIs.

## Discussion and conclusions

In this study, we developed HormoNet that predicts hormone-drug interactions and possible risk level of their interaction. To this end, we took advantage of a deep neural network in consideration of the interaction between hormone receptors and drug targets. AAC is one of the common methods for encoding protein sequences. However, the main challenge of this method is the loss of protein sequence information that can affect the model performance. To overcome this problem, we have applied the PseAAC strategy, because PseAAC has been broadly applied in different studies and has provided sufficient performances in the field of protein interaction predictions [11–19]. Thus, in this study we used this technique to encode protein sequences.

In several studies [20–25], it has been proved that the physicochemical and biochemical characteristics (e.g., hydrophilicity, hydrophobicity, polarity, hydrogen bonds, salt bridges) have an essential role in protein associations. Therefore, we have collected 30 different sequence-based and structural-based features from protein sequences which the use of this large number of properties is unprecedented in this field.

In our study, we had imbalanced problem in our dataset since the classes' distribution were not similar, therefore, we used SMOTE to deal with this problem. According to [18, 26–32], among different methods to handle imbalance problem, SMOTE had superior performances on the biological data. According to Table 2 in the experimental results generated from our predictor, the results on the test dataset were significantly improved after applying the SMOTE.

In this study a deep learning model for the first time has been developed for predicting of HDI. The advancement of interaction prediction in various fields of computational biology can provide valuable insights into genetic markers, related diseases, and ncRNAs related with drug [33–40]. Therefore, future studies in these areas for biological predictions could be performed using machine/deep learning methods. Wang et al. [41] proposed a model named DMFGAM to predict Human ether-a-go-go-related gene blockers based on a fully connected neural network. They used molecular fingerprint features and molecular graph features are fused as the final features of the compounds to make the feature expression of compounds. Sun et al. [42] proposed a model named as graph convolutional network with graph attention network (GCNAT) based on deep learning approaches, for predicting potential metabolic-disease associations. They constructed a heterogeneous network using known associations of metabolite-disease, metabolite-metabolite similarities, and disease-disease similarities. In another study [43], Wang et al. presented a deep learning model named GCNCRF using graph convolutional neural network and conditional random field to predicte human lncRNA-miRNA interactions. They constructed a heterogeneous network based on interactions of lncRNA-miRNA, lncRNA/miRNA similarity network, and the lncRNA/miRNA feature matrix.

Deep Learning as a subfield of machine learning methods have been demonstrated to exhibit unprecedented performance in different biological prediction areas [40, 44–53]. Here, we have proposed a deep neural network model, termed HormoNet, to predict HDI and their risk level.

We compared the MLP, CNN, and LSTM outcomes on our benchmark dataset to develop our prediction model for HDI. The performance of each network and algorithm was determined by assessing how they could correctly predict whether the hormones receptors were interacting with a specific drug target or not.

Figure 1 shows that CNN had superior outcomes compared to MLP and LSTM methods. According to [54–59], CNNs have had more efficient outcomes in biological problems. Generally, CNN have had better performances in classification of image data. In this study, since HDI data are liked 2D images, therefore CNN-1D network had higher performance compare to MLP and LSTM networks.

In order to select the most important features and ranking them we tested three different FS strategies including RF, LSVC, and XGBoost. The 162, 293, and 177 optimal features were chosen for RF, LSVC, and XGBoost, respectively, according to the nature of our dataset and the optimized parameters. The parameters for each algorithm were set based on our several feature selections experiments.

However, according to Fig. 2, the performance of our predictor was reduced after applying FS methods. Which, it can be justify that the deep learning-based methods require large-scale data. The dimension of our initial dataset for HDI was 9230*900 for HDI, which reduced to 9230*162, 9230*293, and 9230*177, for RF, LSVC, and XGBoost, respectively, which decreased our model’s performance.

The dimension of our initial dataset for HDI risk level was 7963*900 which reduced to 7963*334, 7963*337, and 7963*259, for RF, LSVC, and XGBoost, respectively, which decreased our model’s performance. According to the obtained results, it is clear that the predictor’s performance would be higher when fed with larger dataset.

Roc curves have been applied as a common method to evaluate the performance of models based on machine/deep learning methods [60–64]. Thus, we used this method to evaluate the performance of the proposed model in our experiments. It is a better technique instead AUC because AUC considers only numerical values. Figure 4 shows ROC curves for HDI and risk level of HDIs, respectively. The curves illustrate that the algorithm found class 1—level moderate—harder to learn, probably because, the class is highly variable among samples and across time.

In this study, for the first time we have proposed HormoNet, a novel deep learning technique for HDI identification based on physicochemical and conformational properties from hormone and drugs pairs. Moreover, we constructed two novel datasets for HDI and HDI risk levels. In addition, we have proposed a learning approach directed to predict the risk level of HDIs. We have performed several experiments to test the performance of our model. Experimental evaluations indicate that, HormoNet achieved high level of performance on our benchmark datasets regarding accuracy, f1score, precision and recall. This study is unique in three ways: (1) it is the first study that uses deep learning techniques for prediction. (2) In addition to predicting hormone-drug interactions, it also predicts their risk level. (3) We have collected 30 different sequence-based and structural-based features from protein sequences to create our benchmark datasets which the use of this large number of properties is unprecedented in this field.

HormoNet has indicated to be able to provide insights into understanding HDI's nature, which can be helpful for all scientists and researchers in this field.

One of the main challenges of this study was about limitation of the number of databases for hormone-receptor interactions. Another challenge of this study was the lack of a database including hormone-drug interactions and a regarding datasets. Since this study is the first effort in the field of HDI prediction using sequence-based features of hormone receptors and drug targets, therefore, more studies in this area are required by using of other feature extraction strategies. In addition, this study focuses on hormonal drugs, but there are other types of drugs that require more research to focus on hormonal interactions with other types of drugs. A powerful web server for HDI identification can be a very helpful tool for researchers in this field, therefore, future studies by focusing on designing web server for HDI, are recommended. Since the outcomes of proposed model presented the potential of HormoNet along with the use of properties, therefore in other further efforts can use it.

## Methods

**Fig. 5:**
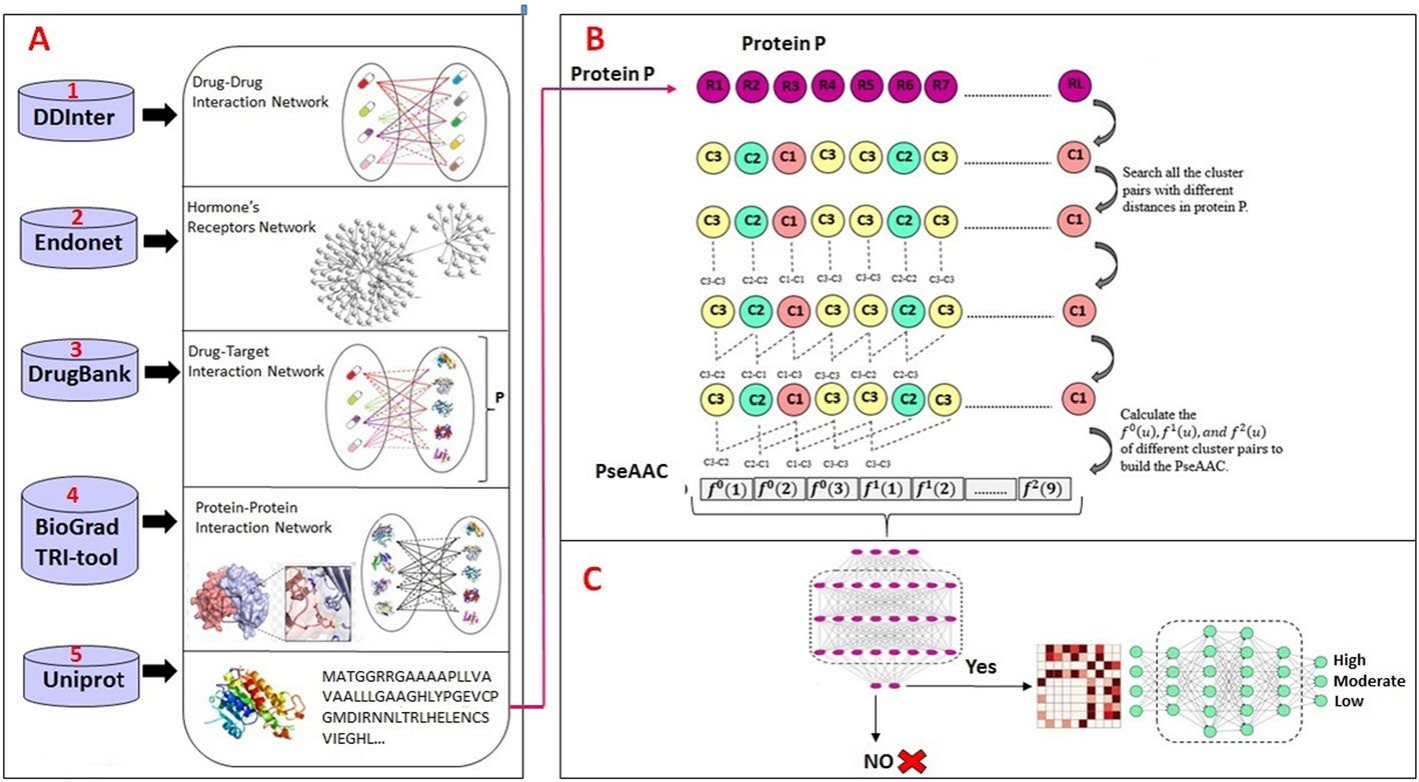
A schematic overview of the training module of HormoNet. Where,** A** presents data gathering processing using 5 different databases; **B** shows feature extraction strategy using PseAAC and constructing our benchmark datasets; **C** displays HDI prediction and their risk level; and HDI is Hormone Drug Interaction

This section presents detailed information of the constructing our datasets, including data gathering, feature extraction, and balancing dataset. Additionally, prediction model construction and model evaluation have been elaborated. It should be noted that, all technique in this study implemented in Python language using Python 3.8.16 version. To implement the deep learning methods Keras library of Python was used in Google Collaboratory environment. Figure 5 illustrates a schematic overview of the training module for HormoNet.

### Data collection

This study had two stages: first stage, prediction of possible hormone-drugs interactions. Second stage, if there is interaction, prediction of risk levels (major, moderate, and minor) of hormone-drugs interactions. Therefore, we constructed two different datasets for different purposes including, the training dataset of HormoNet for HDI includes interacting and non-interacting hormone-drug pairs. As well, the training dataset of HormoNet for HDI risk levels containing the risk level of those positive interaction in the previous dataset for hormone-drug pairs.

In order to construct a reliable dataset, we collected following data from six different databases:

1. Human endogenous hormones and their receptors from EndoNet [65] 2. Drug-drug interactions from DDInter [66] 3. Drug-target associations from DrugBank [67] 4. Protein sequences from UniProtKB/Swiss-Prot [57–68] **5.** Protein–protein interactions from BioGRID [69] and TRI-tool [70].

**Fig. 6:**
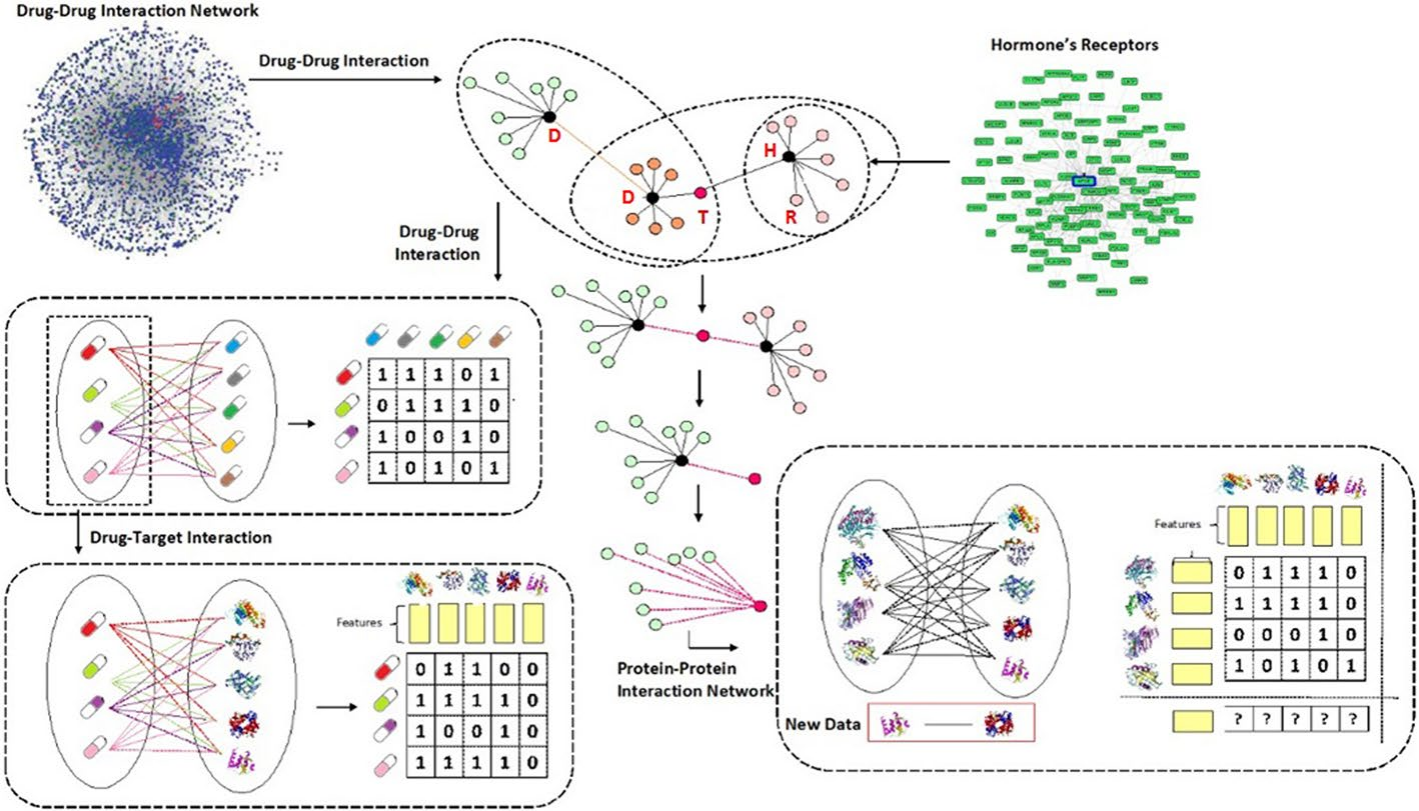
Flowchart of performed methodology to gather data and construct our datasets. Where, D: Drug, H: Hormone, R: Receptor, T: Target

First, we obtained drug-drug interactions and clarified a relation for every drug-drug interaction. Relations have been classified into three categories including: class A: *'risk level of interaction of drug A and drug B is major'*, class B: *'risk level of interaction of drug A and drug B is moderate'*, and class C: *'risk level of interaction of drug A and drug B is minor'*. The ‘*drug A–drug B interaction*’ is extracted if drug A is one of the human hormones, we collected from EndoNet (i.e., from *‘risk level of interaction of drug A and drug B’* to *‘risk level of interaction of hormone A and drug B’*). Second, Hormones and drugs should have at least one protein receptor or protein target, respectively. Thus, we extracted human hormones that have one or more protein receptors and obtained drugs which have one or more protein targets. Next, for the proteins, since the identifiers of protein receptors and targets are presented in DrugBank and EndoNet (e.g., *NR3C1, PPARG, KCNJ1, *etc*.*), therefore, we prepared their sequences by searching in UniProtKB/Swiss-Prot based on the best name matches. It should be noted that, we removed sequences that their length was smaller than *50* or contained *X* in their amino acid sequences (e.g., *CFTR, CACNA1B, PIK3R2*, and etc.). *Then*, we extracted those protein receptors and protein targets that had physical interactions based on their sequences. *Finally*, for constructing the first stage’s dataset, the total number of instances were *9230*, which contain *4773* positive and *4457* negative instances, include *28* hormones, *443* drugs and *28* hormone receptors and *321* protein targets were obtained. For building the second stage’s dataset, the *4773* interactions containing risk levels have been considered include *21* hormone, *20* hormone receptors, *312* drugs and *295* protein targets were obtained (see Fig. 6).

### Hormone–receptor interaction

We obtained hormones that have one or more receptors from EndoNet database. EndoNet contains physical interactions between human hormones and their protein receptors. As a result, *283* human hormones and *451* receptors are extracted.

### Drug–drug interaction

We collected drug-drug interaction from DDInter database. DDInter provides drug-drug associations with their risk levels. It contains different entries of drug-drug interactions, which among them we chose interactions involving hormonal drugs, excluding sex hormones and insulin’s drugs. A total number of *8961* interactions have been found.

### Drug–target interaction

We obtained drug’s protein targets by searching in DrugBank based on the best name matches. DrugBank provides physical interactions between drugs and their specific protein targets. As a result, *2209* drug protein target associations have been found.

### Protein–protein interaction

Physical possible protein proteins are extracted from BioGRID (Biological General Repository for Interaction Datasets) and TRI-Tool (Transcriptional Regulation Interactions) databases. The BioGRID is resource that houses manually curated protein and genetic interactions from multiple species and human. The TRI_tool is a sequence-based tool for protein interactions prediction in the human transcriptional regulation. As a result, *9230* interactions containing *4773* positive and *4457* negative interactions have been obtained.

### Feature construction

In this study, the amino acid composition and pseudo-amino acid composition were used for encoding the protein sequences of each hormone’s receptors and drug’s targets.

### Amino acid composition (AAC)

AAC is a common technique in biological problems for encoding proteins. It calculates the number of amino acids of each type in a protein sequence. For a sequence with *N* amino acids:

1 $${\text{f}}\left({\text{i}}\right)={\text{n}}({\text{i}})/{\text{N}}$$

where *i* is the *20* amino acid residues and *n(i)* is the number of amino acids type *i*.

### Pseudo-amino acid composition (PseAAC)

One of the main challenges in AAC method is losing the information of protein sequences which can affect the prediction performances. To overcome this problem, we have applied PseAAC strategy. The PseAAC algorithm was introduced for the first time in *2001* in molecular biology [71]. It was designed to improve the prediction quality of protein subcellular properties. PseAAC has been used in various biological problems [72–78] for extracting features from proteins. It can be described as follows [79]:

Consider a protein chain *S* with *N* amino acid residues:

2 $${{\text{S}}={\text{R}}}_{1}{{\text{R}}}_{2}{{\text{R}}}_{3}\dots {{\text{R}}}_{{\text{N}}}$$

The order's effect of protein sequence can be approximately reflected with a set of separate correlation factors as defined below:

3 $$\left\{\begin{array}{c}{\uptheta }_{1}=\frac{1}{{\text{L}}-1}\sum\limits_{{\text{i}}=1}^{{\text{L}}-1}\Theta ({{\text{R}}}_{{\text{i}}}, {{\text{R}}}_{{\text{i}}+1})\\ {\uptheta }_{2}=\frac{1}{{\text{L}}-2}\sum\limits_{{\text{i}}=1}^{{\text{L}}-2}\Theta ({{\text{R}}}_{{\text{i}}}, {{\text{R}}}_{{\text{i}}+2})\\ {\uptheta }_{3}=\frac{1}{{\text{L}}-3}\sum\limits_{{\text{i}}=1}^{{\text{L}}-3}\Theta \left({{\text{R}}}_{{\text{i}}}, {{\text{R}}}_{{\text{i}}+3}\right)\\ .\\ .\\ .\\ {\uptheta }_{\uplambda }=\frac{1}{{\text{L}}-\uplambda }\sum\limits_{{\text{i}}=1}^{{\text{L}}-\uplambda }\Theta \left({{\text{R}}}_{{\text{i}}}, {{\text{R}}}_{{\text{i}}+\uplambda }\right), (\uplambda <{\text{L}})\end{array}\right.$$

*θ*_1_,* θ*_2_, …, *θ*_*λ*_ are the *1*-tier, *2*-tier, and *λ*th tier sequence order correlation factors, respectively. The correlation function compared as:

4 $$\Theta \left({{\text{R}}}_{{\text{i}}}, {{\text{R}}}_{{\text{j}}}\right)= \frac{1}{3}\left\{{\left[{{\text{H}}}_{1}\left({{\text{R}}}_{{\text{j}}}\right)-{{\text{H}}}_{1}\left({{\text{R}}}_{{\text{i}}}\right)\right]}^{2}+{\left[{{\text{H}}}_{2}\left({{\text{R}}}_{{\text{j}}}\right)-{{\text{H}}}_{2}\left({{\text{R}}}_{{\text{i}}}\right)\right]}^{2}+{\left[{\text{M}}\left({{\text{R}}}_{{\text{j}}}\right)-{\text{M}}\left({{\text{R}}}_{{\text{i}}}\right)\right]}^{2}\right\}$$

*H*_*1*_*(R*_*i*_*), H*_*2*_*(R*_*i*_*),* and *M(R*_*i*_*)* are, some physicochemical and biochemical attribute values of the amino acid *R*_*i*_*. H*_*1*_*(R*_*j*_*), H*_*2*_*(R*_*j*_*),* and *M(R*_*j*_*)* are the corresponding values of the amino acid *R*_*j*_. The values of each attribute are described from the original values by the following formula:

5 $$\left\{\begin{array}{c}{{\text{H}}}_{1}\left({\text{i}}\right)=\frac{{{\text{H}}}_{1}^{0}\left({\text{i}}\right)-\sum\limits_{{\text{i}}=1}^{20}\frac{{{\text{H}}}_{1}^{0}\left({\text{i}}\right)}{20}}{\sqrt{\frac{\sum\limits_{{\text{i}}=1}^{20}\left[{{\text{H}}}_{1}^{0}\left({\text{i}}\right)-\sum\limits_{{\text{i}}=1}^{20}\frac{{{\text{H}}}_{1}^{0}\left({\text{i}}\right)}{20}\right]}{20}}}\\ {{\text{H}}}_{2}\left({\text{i}}\right)=\frac{{{\text{H}}}_{2}^{0}\left({\text{i}}\right)-\sum\limits_{{\text{i}}=1}^{20}\frac{{{\text{H}}}_{2}^{0}\left({\text{i}}\right)}{20}}{\sqrt{\frac{\sum\limits_{{\text{i}}=1}^{20}\left[{{\text{H}}}_{2}^{0}\left({\text{i}}\right)-\sum\limits_{{\text{i}}=1}^{20}\frac{{{\text{H}}}_{2}^{0}\left({\text{i}}\right)}{20}\right]}{20}}} \\ M\left({\text{i}}\right)=\frac{{{\text{M}}}^{0}\left({\text{i}}\right)-\sum\limits_{{\text{i}}=1}^{20}\frac{{{\text{M}}}^{0}\left({\text{i}}\right)}{20}}{\sqrt{\frac{\sum\limits_{{\text{i}}=1}^{20}\left[{{\text{M}}}^{0}\left({\text{i}}\right)-\sum\limits_{{\text{i}}=1}^{20}\frac{{{\text{M}}}^{0}\left({\text{i}}\right)}{20}\right]}{20}}}\end{array}\right.$$

*H*_*1*_*(i), H*_*2*_*(i),* and *M(i)* are the original values of attributes for the *20* native amino acids. Consequently, for a protein sequence S, the PseAAC is demonstrated via a *(20* + *λ)-* Dimensional vector as below:

6 $${\left[{{\text{V}}}_{1},{{\text{V}}}_{2}, \dots ,{{\text{V}}}_{20}, {{\text{V}}}_{21}, \dots , {{\text{V}}}_{20+\uplambda } \right]}^{{\text{T}}}$$

where *T* is called the transpose operator.

7 $${{\text{X}}}_{{\text{u}}}=\left\{\begin{array}{c}\frac{{{\text{f}}}_{{\text{u}}}}{\sum\limits_{{\text{i}}=1}^{20}{{\text{f}}}_{{\text{i}}}+\upomega \sum\limits_{{\text{j}}=1}^{\uplambda }{\uptheta }_{{\text{j}}}}, (1\le u\le 20)\\ \frac{{\mathrm{\omega \theta }}_{{\text{u}}-20}}{\sum\limits_{{\text{i}}=1}^{20}{{\text{f}}}_{{\text{i}}}+\upomega \sum\limits_{{\text{j}}=1}^{\uplambda }{\uptheta }_{{\text{j}}}}, (20+1\le u\le 20+\lambda )\end{array}\right.$$

where* f*_*i*_ indicates the number of the *20* amino acids, *θ*_*j*_ indicates *j*th tier sequence-correlation factor, and the ω is the weight factor of the effect of sequence order.

The first 20 elements in Eq. 4 indicates the effect of amino acid composition, and the rest of them *(20* + *1 to 20* + *λ)* indicate the sequence-order effect. Therefore, the whole of *20* + *λ* elements is PseAAC. Here, we set *ω* = *0.05* and *λ* = *30*.

In this study, we used *30* physicochemical (i.e., molecular weight, mass, bulkiness, hydrophobicity, hydrophilicity, melting point, transfer-free energy, solvation free energy, buriability, volume, polarity, relative mutability, isoelectric point, amino acid distribution, chromatographic index, residue volume, compressibility, hydration number, Shape, Stability, power to beat the N terminal, C terminal, unfolding entropy change, unfolding enthalpy, unfolding Gibbs free energy change, middle of alpha helix, Alpha-helical tendency, Beta-helical tendency, Turn tendency, and coil tendency) amino acid properties. The 30 properties were reached from [80, 81], which could be found in Additional file 1.

### Balancing the dataset

The class imbalance problem typically refers to a problem with classification problems where the distribution of each classes is not similar and equal. Consequently, it can limit the performance of the model because the model tends to be overwhelmed by the majority classes and ignore the small ones [82]. In our study we had imbalanced problem in our dataset since the classes' distribution were not similar, therefore, we used SMOTE to deal with this problem. we applied SMOTE to deal this problem [10]. According to [18, 26–32], among different methods to handle imbalance problem, SMOTE had superior performances on the biological data. SMOTE is an over-sampling technique for balancing dataset in which the minority class is over-sampled by generating “synthetic” instances rather than by over-sampling with replacement [83]. New synthetic samples are generated for each minority class until all classes reach a balanced number equal to the number of the majority class's samples. Our constructed datasets are available in Additional files 2 and 3.

In SMOTE the synthetic data generation is based on a k-nearest neighbor’s algorithm and linear interpolation [84]. Take *u* is a random number between *0* and *1*; *x* is the feature vector (instance) under consideration of the minority class and (*x*^*R*^) is its nearest neighbor. The SMOTE instances are linear combinations of two similar instances from the minority class (*x* and *x*^*R*^) and are determined as

8 $${\text{s}}={\text{x}}+{\text{u}}.({{\text{x}}}^{{\text{R}}}-{\text{x}})$$

**Fig. 7:**
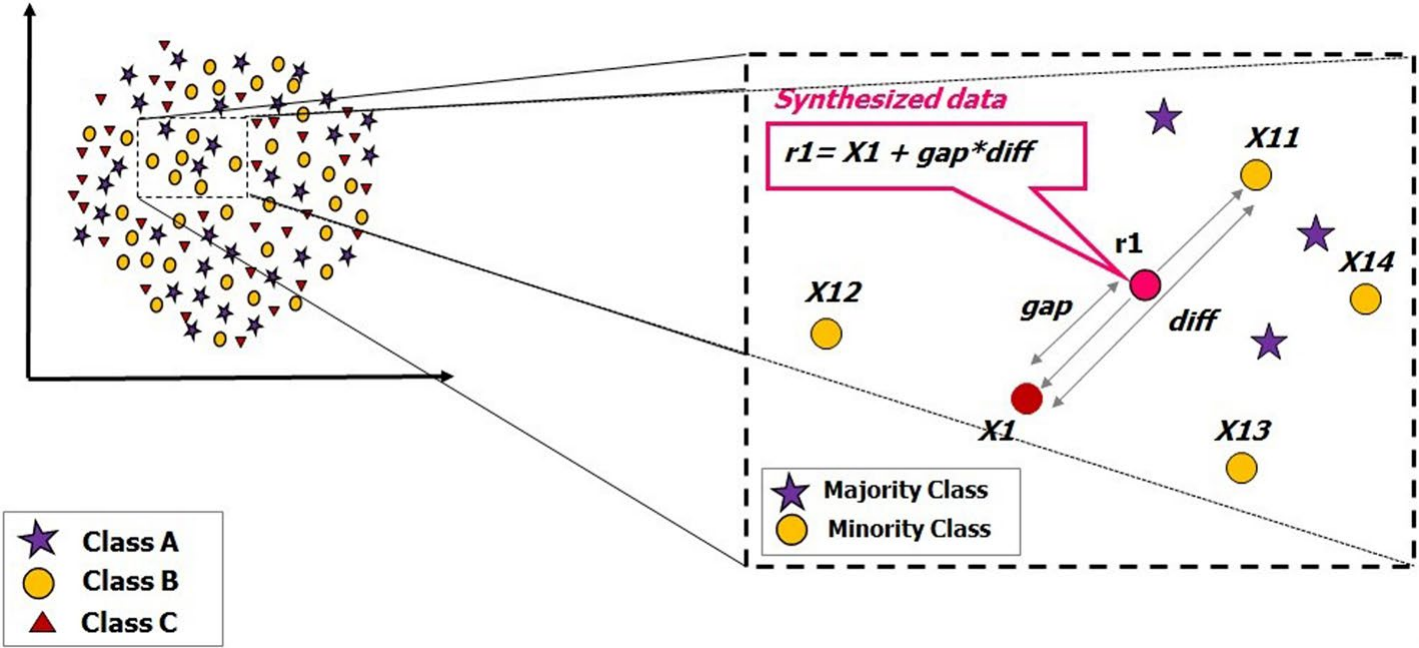
Illustration of SMOTE technique in order of imbalance handling and generating synthesized data

The synthetic instance will be at a random point along the line segment between two specific features. This technique effectively forces the decision-making regions of the minority class instances to become more general. Figure 7 describes this procedure.

### Description of deep neural network model

In this study, we have selected a 1-Dimensional Convolutional Neural Network (1D-CNN; [49]) as our predictor.The proposed *1*D-CNN model for prediction of HDI is composed of an input layer, four convolutional layers, two pooling layers, one fully connected (FC) layer, and a sigmoid output layer. The proposed *1D-CNN* model for prediction of risk level of HDI pairs is composed of an input layer, four convolutional layers, two pooling layers, two batch size normalization layers, one activation layer, five fully connected layer, and a categorical cross entropy soft-max output layer.

A one dimensional convolutional operation can be determined as [85]:

9 $${y}_{j}^{l}=b\left(\sum\limits_{i=1}^{{N}_{l-1}}conv1D\left({w}_{i,j}^{l},{x}_{i}^{l-1}\right)+{b}_{j}^{l}\right)$$

where ${y}_{j}^{l}$ indicates the *j*th feature map in the layer *l*; ${w}_{i,j}^{l}$ indicates the trainable convolutional kernel; ${x}_{i}^{l-1}$ indicates the *i*th feature map in the layer *(l–1)*; conv1D indicates the 1D convolution operation without zero-padding, ${N}_{l-1}$ indicates the number of feature maps in the layer *(l–1)*; ${b}_{j}^{l}$ indicates the bias of the *j*th feature map in the layer *l*; and *b* is an activation function named rectified linear unit (Relu) for avoiding the over-fitting problem. It is determined as

10 $${\text{ReLU}}\left({\text{x}}\right)=\left\{\begin{array}{c}x, x\ge 0\\ 0, x<0\end{array}\right.$$

Then, 64 feature maps with the size of 176 × 1 are outputted and then passed through a max-pooling layer. It is calculated as:

11 $${{\text{p}}}_{{\text{i}}}^{{\text{a}}}=max({{\text{p}}}_{{\text{i}}}^{{{\text{a}}}^{{{\prime}}}};{\text{a}}\le {{\text{a}}}^{{{\prime}}}<{\text{a}}+{\text{s}})$$

where ${p}_{i}^{{a}{\prime}}$, ${p}_{i}^{a}$, and *s* are the *a′*th neuron in the *i*th feature map before and after max-pooling operation, the size of pooling window, respectively.

In this study, the size of pooling window and the stride of windows were set *2* for Pooling Layer *1*. Which it can sufficiently reduce the parameters' training number in the predictor and accelerate the process of training. The outputs of the pooling operation are 64 feature maps with the size of *88* × *1*. Then, Conv Layer *3* and Conv Layer *4* are followed for extracting higher-level features which can facilitate the classification. There are *128* and *1024* kernels in the shape of *3* × *1* in the Conv Layer *3* and Conv Layer *4*, respectively. ReLU function was applied for non-linear activation. After passing the feature maps through all 1D convolutional layers, the 1024 feature maps with the size of 82 × 1 were obtained. They were fed into GlobalAveragePooling1D operation with 256 neurons. Then, dropout was applied to the output of the pooling layer for alleviating the over-fitting problem.

The output features were fed into four fully connected layers with 128 neurons. Finally, a Sigmoid and Softmax output layers were added to the proposed model for HDI and risk level final recognition, respectively.

The last layer's output for risk level classifier was acquired using the Softmax function. It is described as:

12 $${\hat{\text{y}}}_{{\mathbf{i}}} = {\text{argmax}}\left( {\frac{{{\mathbf{e}}^{{{\mathbf{y}}_{{\mathbf{i}}} }} }}{{\mathop \sum\limits \limits_{{{\mathbf{i}} = 1}}^{5} {\mathbf{e}}^{{{\mathbf{y}}_{{\mathbf{i}}} }} }}} \right)$$

The last layer's output for HDI classifier was acquired using the sigmoid function. It is calculated as:

13 $${\text{Sigmoid}}\left({\text{x}}\right)=\left(\frac{1}{1+{{\text{e}}}^{-{\text{x}}}} \right)$$

For prediction of the risk levels of each HDI, the optimization of the parameters of the model was based on the categorical cross-entropy loss function. It is described as follows:

14 $${\text{loss}} = - \mathop \sum\limits \limits_{{{\text{i}} = 1}}^{5} \left( {{\text{y}}_{{\text{i}}}^{*} \cdot {\text{log}}\,{\hat{\text{y}}}_{{\text{i}}} } \right)$$

where ${{\text{y}}}_{{\text{i}}}^{*}$ is the corresponding target value (*1* for the correct class and *0* incorrect class) and ${\hat{\text{y}}}_{{\text{i}}}$ is *i*th output prediction.

The rmsprop algorithm [86] was applied for optimizing the model by updating the parameters of the model.

For HDI prediction we used binary cross-entropy cost function. It is described as follows:

15 $${\text{E}}= -\frac{1}{{\text{n}}}\sum\limits_{{\text{i}}}\sum\limits_{{\text{d}}}\left[{\text{vIna}}+(1-{\text{v}}){\text{In}}(1-{\text{g}})\right]$$

where *i* is the index of training sample, *v* is the true value of sample *i*, which its value can be *0* or *1*, *g* is the predicted network’s output for *0* or* 1* value of sample *i*, and *d* is the different labels index. Consequently, the value of *E* will get less if the predicted results are close to the true values. Thus, to get the most optimal performance, the function must be minimized since the cross-entropy is a non-negative function. The final optimized values are as follow: Epochs = 50, Learning rate = 0.00025, and batch size = 16.

### Performance evaluation

Here, we applied threefold cross-validation strategy for evaluation of our predictor's performance. In this method, the whole dataset is randomly separated into 3 folds which two-folds are applied for training and one for testing. This technique is repeated three times and each sample is tested once. To evaluate the performance of our model, we used four metrics include accuracy, precision, recall, and F1-score which were determined as follow:

16 $${\text{Accuracy}}=\frac{{\text{TP}}+{\text{TN}}}{{\text{TP}}+{\text{TN}}+{\text{FP}}+{\text{FN}}}$$

17 $${\text{Precision}}= \frac{{\text{TP}}}{{\text{TP}}+{\text{FP}}}$$

18 $${\text{Recall}}= \frac{{\text{TP}}}{{\text{TP}}+{\text{FN}}}$$

19 $${\text{F}}1\text{ score}= \frac{{\text{Precision}}\times \mathrm{ Recall}}{{\text{Precision}}+{\text{Recall}}}$$

where for a given class, the values of true positives (TP) and false negatives (FN) display the number of samples of the class that are predicted by the model correctly classified and incorrectly classified, respectively. Also, true negatives (TN) and false positives (FP) display the number of samples not belonging to the class that are correctly predicted as non-belonging to the class and the number of samples not belonging to the class that are incorrectly classified as belonging to the class, respectively.

### Supplementary Information

**Additional file 1.** Selected amino acids’ properties.

**Additional file 2.** Hormone-drug interaction dataset.

**Additional file 3.** Hormone-drug interaction risk level dataset.

## Data Availability

Our benchmark datasets and the source codes for HormoNet are available in: https://github.com/EmamiNeda/HormoNet.
